# Comparison of fatalities due to COVID-19 and other nonexternal causes during the first five pandemic waves: Results from multiple cause of death statistics in Bavaria

**DOI:** 10.1007/s00103-024-03914-5

**Published:** 2024-07-16

**Authors:** Andrea Buschner, Katharina Katz, Andreas Beyerlein

**Affiliations:** 1 Bavarian State Office for Statistics, Division: Population Statistics and Demography, Fürth, Germany; 2 Bavarian Health and Food Safety Authority, State Institute for Health II - Task Force for Infectious Diseases Infectious Disease Epidemiology, Surveillance and Modelling Unit (GI-TFI2), Oberschleißheim, Germany

**Keywords:** SARS-CoV-2, Comorbidities, Mortality, Wave-specific causes of death, Preexisting diseases, SARS-CoV-2, Komorbiditäten, Mortalität, Wellenspezifische Todesursachen, Vorerkrankungen

## Abstract

**Background:**

Older age is a risk factor for a fatal course of SARS-CoV‑2 infection, possibly due to comorbidities whose exact role in this context, however, is not yet well understood. In this paper, the characteristics and comorbidities of persons who had died of COVID-19 in Bavaria by July 2022 are shown and compared with the characteristics of other fatalities during the pandemic.

**Methods:**

Based on data from multiple cause of death statistics, odds ratios for dying from COVID-19 (compared to dying from other nonexternal causes of death) were calculated by using logistic regression models, stratified by age, sex, and pandemic waves.

**Results:**

In Bavaria, a total of 24,479 persons (6.5% of all deaths) officially died from COVID-19 between March 2020 and July 2022. In addition to increasing age and male sex, preexisting diseases and comorbidities such as obesity, degenerative diseases of the nervous system, dementia, renal insufficiency, chronic lower respiratory diseases, and diabetes mellitus were significantly associated with COVID-19–related deaths. Dementia was mainly associated with increased COVID-19 mortality during the first and second waves, while obesity was strongly associated during the fourth wave.

**Discussion:**

The frequency of specific comorbidities in COVID-19 deaths varied over the course of the pandemic. This suggests that wave-specific results also need to be interpreted against the background of circulating virus variants, changing immunisation levels, and nonpharmaceutical interventions in place at the time.

**Supplementary Information:**

The online version of this article (10.1007/s00103-024-03914-5) contains supplementary material, which is available to authorized users.

## Abstract (de)

**Hintergrund:**

Ein höheres Alter stellt einen Risikofaktor für einen tödlichen Verlauf einer SARS-CoV-2-Infektion dar, möglicherweise bedingt durch Komorbiditäten, deren genaue Rolle in diesem Kontext jedoch noch nicht gut verstanden ist. Im vorliegenden Beitrag werden Charakteristika sowie Komorbiditäten der bis Juli 2022 in Bayern an COVID-19 Verstorbenen im Pandemieverlauf aufgezeigt und mit den Merkmalen anderer Verstorbener verglichen.

**Methoden:**

Basierend auf Daten der amtlichen Todesursachenstatistik wurden mit Hilfe logistischer Regressionsmodelle Odds-Ratios für das Versterben an COVID-19 (im Vergleich zum Versterben an anderen natürlichen Todesursachen) stratifiziert nach Alter, Geschlecht und Pandemiewellen berechnet.

**Ergebnisse:**

In Bayern verstarben von März 2020 bis Juli 2022 offiziell insgesamt 24.479 Personen (6,5 % aller Sterbefälle) an COVID-19. Neben zunehmendem Alter und männlichem Geschlecht waren Vor- und Begleiterkrankungen wie Adipositas, degenerative Erkrankungen des Nervensystems, Demenz, Niereninsuffizienz, chronische Erkrankungen der unteren Atemwege und Diabetes mellitus signifikant mit COVID-19-bedingtem Versterben assoziiert. Demenz war hauptsächlich in der ersten und zweiten Welle, Adipositas besonders stark während der vierten Welle mit erhöhter COVID-19-Sterblichkeit assoziiert.

**Diskussion:**

Die Häufigkeit bestimmter Komorbiditäten bei Personen, die an COVID-19 verstorben sind, variierte im Pandemieverlauf. Dies deutet darauf hin, dass wellenspezifische Ergebnisse auch vor dem Hintergrund zirkulierender Virusvarianten, sich verändernder Immunisierungsgrade und der zum jeweiligen Zeitpunkt geltenden Schutzmaßnahmen interpretiert werden müssen.

## Introduction

At the end of January 2020, the first case of severe acute respiratory syndrome coronavirus type 2 (SARS-CoV-2) infection in Germany was confirmed [1]. The subsequent waves of the COVID-19 pandemic were characterised by different levels of infection numbers, hospitalisation rates, and death rates [2]. This was largely due to changing immunity levels following infections, COVID-19 vaccinations that became available at the end of 2020 [3], and the temporary dominance of different virus variants [4]. The first two waves were dominated by the wild type, whereas in the third, fourth, and fifth waves, the alpha, delta, and omicron variants were predominant, respectively [2]. The later variants were characterised by gradually increasing transmissibility rates. At the same time, the risk of a severe COVID-19 course in infected cases was highest for the delta variant and lowest for the omicron variant [5]. In addition, the relevance of certain exposure sites such as the home environment, the workplace, and day-care and senior citizen facilities changed between the waves, also depending on the respective infection protection measures in force [6, 7]. Therefore, certain age groups were affected by the burden of infection to varying degrees in the different disease waves [4]. Besides male sex, older age has frequently been found to be a risk factor for a severe or fatal course of COVID-19 [8–11]. Comorbidities associated with older age are suspected as a possible reason. For example, chronic respiratory diseases [12], kidney diseases [13], and obesity [14] could be identified as possible risk factors for a severe course of the disease.

However, previous studies were often limited with respect to their sample size and/or the recording of comorbidities, or they covered only a relatively short period of the pandemic. In this study, we used data from multiple cause of death statistics to describe the case numbers of all persons residing in Bavaria who died of COVID-19 during the first five pandemic waves from March 2020 to July 2022. We further compared their characteristics and comorbidities with those who died of other nonexternal causes during the same period. During the pandemic, Bavaria was among the federal states of Germany most affected by increased death rates [15].

## Methods

### Data

The following analyses are based on the individual data of the Bavarian cause of death statistics from January 2020 to July 2022. The cause of death statistics are derived from the death certificates that are filled out by the medical certifier during the postmortem examination and subsequently sent to the local health authorities (Table 1). The certifiers are licensed physicians and are advised to provide a conclusive causal chain starting from the immediate cause of death (line Ia) to the preceding cause (line Ib) to the underlying cause (line Ic). According to the definition of the World Health Organization (WHO), the underlying cause is defined as “the disease or injury which initiated the train of morbid events leading directly to death, or the circumstances of the accident or violence which produced the fatal injury” [16]. In the German federal statistical offices, the underlying cause that appears in the official cause of death statistics is determined for each deceased person on the basis of all noted comorbidities. During this coding process, the rules of the WHO are applied with the aid of an automated coding software. Usually—but not necessarily, as is often assumed—the underlying cause is identical to the illness named in field Ic of the death certificate. The coding procedure based on the WHO rules leads to internationally comparable underlying causes.

**Table 1:** Medical part of the Bavarian death certificate

Line			Time interval from onset to death
*Ia*	Immediate cause: disease or condition directly leading to death		
*Ib*	Previous cause: diseases that caused the immediate cause of death		
*Ic*	Underlying cause*		
*II*	Other significant conditions contributing to death		
Epicrisis: further information on the causes of death			

*The underlying cause is defined as “(a) the disease or injury which initiated the train of morbid events leading directly to death, or (b) the circumstances of the accident, or violence which produced the fatal injury” (World Health Organization definition [16]). Source: own table based on the Bavarian death certificate

In addition to the causal chain leading to death in Part I of the death certificate, certifiers can indicate further significant concomitant or preexisting diseases in Part II below the underlying cause [17]. The resulting multicausal chain consisting of these comorbidities, the underlying cause, and the resulting consequences and complications (entered on the death certificate above the underlying cause) has been available for analyses in Bavaria since 2020.

### Statistical methods

Descriptive analyses show the course of COVID-19 fatalities as well as their age and sex distribution during the first two and a half years of the pandemic. The pandemic waves were defined according to the retrospective phase classification of the Robert Koch Institute [2]. In addition, robustness analyses were carried out with an alternative classification of the pandemic phases in which calendar weeks heavily affected by COVID-19 mortality were included. Furthermore, the most frequent comorbidities associated with COVID-19 deaths were compared with those of other nonexternal deaths. A nonexternal death is defined as a death due to illness or old age and one that was not self-inflicted or caused by other people. Here, all diseases and diagnoses listed in the death certificate in Part II or (formally incorrect) in Part I below the underlying cause were considered as comorbidities.

In order to mutually adjust for the various factors associated with COVID-19 mortality, logistic regression analyses were used to calculate odds ratios for dying from COVID-19 (compared to dying from another nonexternal cause of death). In addition to the most frequent comorbidities, obesity [14] and degenerative diseases of the nervous system (including Alzheimer’s disease [18]) were taken into account, since these were also found to be associated with a severe course of COVID-19 in previous research. Besides wave-specific calculations, subgroup analyses were performed for sex and age. Additionally, average marginal effects were calculated for the included predictors in order to exclude possible bias due to unobserved heterogeneity in the groups. All analyses were carried out with SAS Enterprise Guide 7.1.

## Results

### Descriptive analyses

According to the official cause of death statistics, 24,479 people (6.5% of all deaths)—12,916 men (52.8%) and 11,563 women (47.2%)—died due to COVID-19 in Bavaria from March 2020 to July 2022. Another 5579 people died “with” COVID-19, which means that COVID-19 was recorded as a comorbidity on their death certificate without being the underlying cause.

**Fig. 1:**
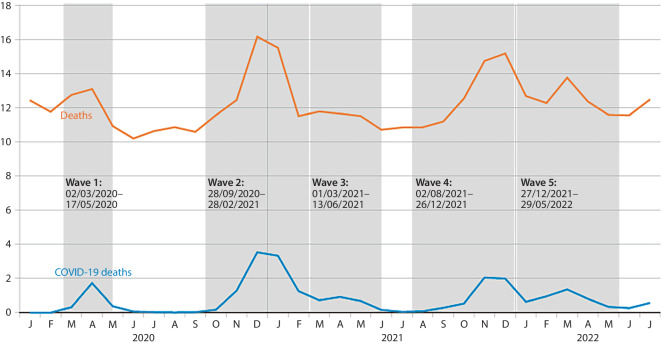
Absolute number of deaths in thousands (including all nonexternal and external causes of death; orange) and number of COVID-19 deaths in thousands (blue) for January 2020 to July 2022 in Bavaria according to the official cause of death statistics. The pandemic waves were defined following the retrospective phase classification of the Robert Koch Institute [2]. Source: own figure

In December 2020 and January 2021, the highest numbers of monthly COVID-19 deaths occurred. During this time period, a total of 3531 and 3329 people, respectively, died due to COVID-19 (almost 22% of monthly deaths; Fig. 1). High numbers of COVID-19 fatalities were also recorded at the end of 2021, with 2048 deaths in November and 1988 in December (13.9% and 13.1% of monthly deaths, respectively).

**Fig. 2:**
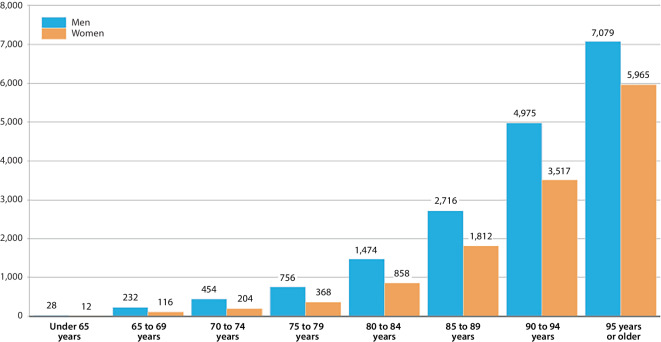
Age-specific COVID-19 mortality rates for March 2020 to July 2022 in Bavaria (per 100,000 inhabitants in the corresponding age/sex group) according to the official cause of death statistics. Source: own figure

The age-specific mortality rates (Fig. 2) indicated that COVID-19 mortality predominantly affected older or very old people of both sexes, whereas across all age groups, men died proportionally more often of COVID-19 than women did. In the oldest age group (95 years or older), a total of 7079 men per 100,000 male residents and 5965 women per 100,000 female residents succumbed to COVID-19 during the observation period.

Those who died of COVID-19 were on average 81.2 years old (standard deviation [SD]: 11.1) and significantly older (t-test: *p* < 0.0001) than others who died of nonexternal causes (mean: 79.5 years; SD: 13.0). Men who died of COVID-19 were on average 78.9 years old (median: 81; SD: 11.2), while women were significantly older at an average of 83.7 years (median: 85; SD: 10.3).

**Fig. 3:**
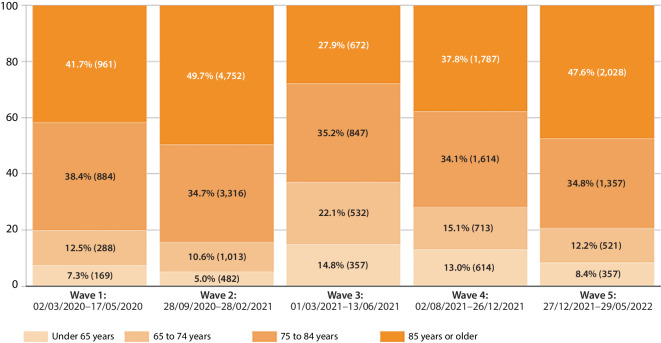
Age distribution of COVID-19 deaths (in percentages and absolute numbers) in Bavaria according to the official cause of death statistics, stratified by pandemic waves following the retrospective phase classification of the Robert Koch Institute [2]. Source: own figure

A wave-specific examination of the age distribution revealed clear differences between the phases of the pandemic. For example, the proportions of COVID-19 deaths in individuals aged 85 years or older in waves one (41.7%), two (49.7%), and five (47.6%) were significantly higher than in waves three (27.9%) and four (37.8%). Accordingly, the third and fourth waves were characterised by higher proportions of younger deceased (Fig. 3).

**Fig. 4:**
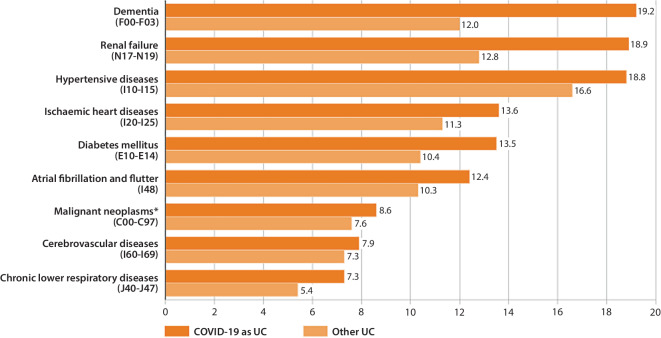
Most frequent comorbidities and previous diseases of persons ≥ 65 years with underlying cause (UC) COVID-19 compared to other nonexternal causes of death between March 2020 and July 2022 in Bavaria (in percent) according to the official cause of death statistics. *Asterisk* indicates lack of secondary neoplasm. Source: own figure.

Due to the comparatively high age of those persons who died from COVID-19, we restricted the descriptive comparisons of comorbidity frequencies between groups to persons aged 65 years or older. The most common comorbidity in people in this age group who died of COVID-19 was dementia (19.2%), compared to 12.0% in those who died of other nonexternal causes. Renal failure was noted on the death certificate in 18.9% of COVID-19 decedents and in 12.8% of the comparison group. Chronic lower respiratory diseases (including chronic obstructive pulmonary disease [COPD]) appeared on 7.3% of the death certificates of COVID-19 decedents and on 5.4% of those of the comparison group. The other comorbidities that were examined in the present analysis (hypertension, ischaemic heart disease, diabetes mellitus, atrial fibrillation and flutter, malignant neoplasms, and cerebrovascular diseases) also occurred significantly more frequently in COVID-19 decedents than in other nonexternal deaths (chi-square test: *p* < 0.001 in each case), even though they showed smaller relative differences between the comparison groups (Fig. 4).

Obesity was not one of the most frequent contributing or preexisting diseases, but it was noted significantly more often on the death certificates of COVID-19 decedents than on those with other nonexternal deaths (1.8% compared to 1.0%; chi-square test: *p* < 0.0001). The same applies to degenerative diseases of the nervous system, which were mentioned as comorbidities in 2.0% of those who died of COVID-19 and in 1.0% of the comparison group.

### Multivariable analyses

**Fig. 5:**
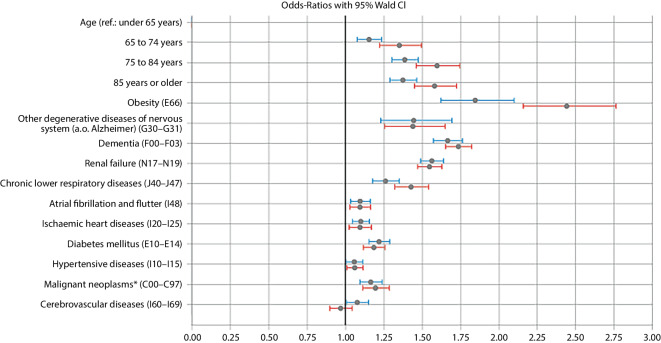
Mutually adjusted odds ratios with 95% confidence intervals for dying from COVID-19 compared to dying from another nonexternal cause of death for men (blue) and women (red) in Bavaria (March 2020—July 2022) according to the official cause of death statistics. *Asterisk* indicates lack of secondary neoplasm. Source: own figure

Logistic regression models with mutual adjustment (Fig. 5) as well as sex- and age-specific stratification (electronic supplementary material, Table S1) showed significant associations for male sex and older age with COVID-19-related death. A wave-specific analysis revealed that age was not consistently associated with COVID-19 as the underlying cause of death in the third and fourth waves (electronic supplementary material, Table S2).

Persons who died of COVID-19 were significantly more often obese; had renal insufficiency, chronic lower respiratory disease, or diabetes mellitus; and, except in the third wave, had dementia or degenerative diseases of the nervous system.

Obesity was more strongly associated with COVID-19 mortality in women than in men, and most strongly in persons under 65 years. Although the association between obesity and COVID-19 mortality was high throughout the whole study period, the fourth wave showed a particularly high proportion of people with obesity among COVID-19 fatalities.

In contrast, dementia as a contributing or preexisting cause was most strongly associated with COVID-19 mortality in the first, second, and fifth waves.

Across all waves, the relevance of renal failure as a comorbidity was higher for COVID-19 decedents than for those with other nonexternal causes of death. A subgroup comparison by age of decedents showed significant differences. The strongest associations between COVID-19 as the underlying cause of death and renal failure were observed in the age groups below 75 or below 65 years, respectively.

For malignant neoplasms, the strongest associations with COVID-19 mortality were also found in the age groups below 65 and between 65 and 74 years. Strikingly high proportions of deceased persons with malignant neoplasms occurred in the fifth wave, i.e. at the beginning of 2022.

Less pronounced sex-, age-, or wave-specific associations were found for the other comorbidities examined.

Further analyses showed that our main findings were neither driven by changing frequencies over time of the most common comorbidities in persons who died from other causes than COVID-19 nor affected by alternative classifications of the pandemic waves (electronic supplementary material, Tables S3–6). Further, average marginal effects analyses (electronic supplementary material, Tables S7 and S8) showed associations that were similar to those from the odds ratios. For example, the probability of a death record with COVID-19 as the cause of death was five percentage points higher in persons who had obesity registered as a comorbidity in their death certificate than for those who had not. This association was particularly strong during the fourth wave, with more than eight percentage points difference.

## Discussion

According to the official cause of death statistics, a total of 24,479 persons, or 6.5% of all deceased, died of COVID-19 in Bavaria from March 2020 to July 2022. Men succumbed to COVID-19 proportionally more often than women in all age groups, relative to other nonexternal causes of death. Overall, older persons had a much higher COVID-19 mortality than younger population groups.

According to our analyses, higher age, male sex, and certain comorbidities such as obesity, degenerative diseases of the nervous system (especially Alzheimer’s disease), dementia, renal insufficiency, chronic diseases of the lower respiratory tract, and diabetes mellitus occurred significantly more often in individuals with COVID-19–related deaths than in those with other nonexternal deaths. While all these comorbidities are known to increase an individual’s mortality risk in general, this analysis of the official multiple cause of death statistics enabled us to show that they are even more prevalent in COVID-19–related deaths than in other nonexternal deaths.

Our findings compare well with those from a large number of meta-analyses [19–23]. A retrospective cohort study conducted in 2020 on 1904 COVID-19 patients from German hospitals pointed to the risk factors of age, male sex, and previous lung diseases [24]. Elsewhere, COPD and chronic kidney disease were identified as important risk factors for increased mortality in COVID-19 patients, consistent with our findings [12, 25]. According to a study from northern Italy, COPD-related mortality increased during the first year of the pandemic due to a high number of fatalities with COVID-19 as the underlying cause of death and COPD as a comorbidity. With the start of the vaccination campaign at the beginning of 2021, COPD-related deaths declined in Italy [26]. A comparison of hospitalised COVID-19 and influenza patients [25] showed that the most common comorbidities in both groups, such as hypertension, diabetes mellitus, chronic kidney disease, and COPD, were noted slightly more often in influenza patients but were associated with a higher risk of severe or fatal disease progression in COVID-19 patients. Results on the vulnerability of specific population groups were used by the Standing Committee on Vaccination (STIKO) for defining the German vaccination strategy, including the prioritisation of certain groups, as well as for the review of these processes [27].

Our wave-specific analyses revealed that persons who died of COVID-19 during the first and second waves (until February 2021) were significantly older than those in the following period. Consistent with this finding, a study from 2021 showed an increased case fatality rate between November 2020 and January 2021 in Germany. Using a decomposition analysis, the authors were able to attribute this to the increased age of the persons who died with a confirmed SARS-CoV‑2 infection [28]. We could show that during the third and fourth waves (i.e., between the beginning of March and mid-June and between the beginning of August and the end of December 2021), the rates of COVID-19–related deaths were comparatively higher in younger people under the age of 65 or 75 years, respectively. This is in line with findings from the first report of the German COVID-19 Autopsy Registry, which observed significantly younger COVID-19 decedents in the third wave as well [29]. We further observed that a very high proportion of COVID-19 decedents had dementia during the second wave. Together with the high age of the deceased in this pandemic phase, this indicates a high mortality in institutions such as nursing homes and homes for the elderly. Other studies support this hypothesis. For example, the number of outbreaks in nursing or old people’s homes in Bavaria more than tripled from the first to the second pandemic wave, and the number of outbreak cases more than quintupled [30]. As a consequence, more than half of the deaths during the second pandemic wave were associated with outbreak cases in facilities for older persons [6]. In the course of the third and fourth waves, in which the COVID-19 deceased were on average significantly younger than at the beginning of the pandemic [31], obesity was noted on death certificates in a strikingly high frequency [32–34]. This is in line with a meta-analysis that reported associations between obesity and severe disease progression or a higher mortality risk following SARS-CoV‑2 infections [14].

It appears likely that these wave-specific associations reflect varying conditions over the course of the pandemic in Germany. First, the presence of different virus variants and the validity or easing of nonpharmaceutical interventions such as lockdowns, school or restaurant closures, contact restrictions, or mask requirements may have been relevant for infection figures and hospitalisation or death rates [35]. Second, changing immunity levels of different population groups due to previous infections or vaccinations [36, 37] may have led to a changing age structure and thus to a changing relevance of certain comorbidities for COVID-19 mortality in the course of the pandemic.

### Limitations

Some methodological limitations should be noted. The quality of cause of death statistics depends on the quality of the data collection [17]. The accuracy of the information provided by the certifier—both with regard to the diagnoses and the correct place of notation on the death certificate—contributes significantly to a higher quality of the statistics. For example, high multimorbidity and insufficient knowledge of the medical history of the deceased may cause difficulties for the certifier when establishing a plausible and unambiguous causal chain. We can therefore not exclude the possibility that relevant comorbidities were overlooked in certain cases. On the other hand, it could also be possible that medical certifiers may have put more effort into the documentation of conditions related to death when the immediate cause of death was COVID-19, thus inducing higher numbers of reported comorbidities in COVID-19–related deaths. It should further be noted that the proportions of comorbidities described here do not indicate disease prevalences in the general population, as only those comorbidities considered to have contributed to death are to be recorded. This difference is particularly striking in the case of obesity, which is known to affect around 20% of the adult population in Germany [38], but it was recorded in only 1% to 2% of the death certificates analysed here. Indeed, although obesity is known to be a risk factor for diseases such as diabetes mellitus and cardiovascular disease, which in turn increase mortality risk, obesity may often not be considered a condition directly associated with death and seems not to be relevant for certain causes of death—such as behavioural, neurological, and accidental causes—anyway [39, 40]. Despite these methodological limitations, the implementation of the multiple cause of death statistics, which have been available in Bavaria since 2020, has opened up additional possibilities for evaluating mortality data and thus offers great potential in cause-of-death-specific mortality research.

In summary, our investigations of the multiple cause of death statistics for Bavaria for the period March 2020 to July 2022 suggest that people who died from COVID-19 were characterised by an increased age and by higher proportions of male sex and certain comorbidities on average, compared to persons who died from other nonexternal causes. However, the relevance of these factors varied to some extent over the course of the pandemic, possibly due to the temporary dominance of different virus variants, immunisation levels, and nonpharmaceutical interventions in place at the time.

## Supplementary Information

The supplementary material contains the comprehensive regression models, which are stratified by gender, age and pandemic waves. It further provides results of various robustness analyses (alternative classification of the pandemic phases, average marginal effects).

## Data Availability

The data of the cause of death statistics are published in aggregated form at https://www.statistik.bayern.de/statistik/bildung_soziales/gesundheitswesen/index.html. The individual data cannot be made publicly available due to data protection reasons.

## Declarations

Since the present study consists of a retrospective analysis of anonymised data, the verdict of an ethics committee was not required for the study. For the studies listed, the ethical guidelines given in each case apply.
