# Erratum to: Comparison of fatalities due to COVID-19 and other nonexternal causes during the first five pandemic waves

**DOI:** 10.1007/s00103-024-03941-2

**Published:** 2024-08-27

**Authors:** Andrea Buschner, Katharina Katz, Andreas Beyerlein

**Affiliations:** 1 Bavarian State Office for Statistics, Division: Population Statistics and Demography, Fürth, Germany; 2 Bavarian Health and Food Safety Authority, State Institute for Health II - Task Force for Infectious Diseases Infectious Disease Epidemiology, Surveillance and Modelling Unit (GI-TFI2), Oberschleißheim, Germany

**Erratum to:**

**Bundesgesundheitsbl 2024**

10.1007/s00103-024-03914-5

In this article, the order that the authors appeared in the author list was incorrect. It should read: Andrea Buschner · Katharina Katz · Andreas Beyerlein.

The original article has been corrected.

