# Supplementary material for: Comparison of fatalities due to COVID-19 and other nonexternal causes during the first five pandemic waves: Results from multiple cause of death statistics in Bavaria
Source: Bundesgesundheitsblatt Gesundheitsforschung Gesundheitsschutz. 2024 Jul 16;67(8):939–46. doi: 10.1007/s00103-024-03914-5 (PMC11282133; doi:10.1007/s00103-024-03914-5)
Supplement: Supplementary file 1 — The supplementary material contains the comprehensive regression models, which are stratified by gender, age and pandemic waves. It further provides results of various robustness analyses (alternative classification of the pandemic phases, average marginal effects). [file 103_2024_3914_MOESM1_ESM.docx]

**Comparison of fatalities due to COVID-19 and other non-external causes during the first five pandemic waves.**

**Results from the multiple cause of death statistics in Bavaria**

Andrea Buschner^1^, Katharina Katz^2^, Andreas Beyerlein^2^

^1^ Bavarian State Office for Statistics, Division: Population Statistics and Demography, Fürth, Germany

^2^ Bavarian Health and Food Safety Authority, State Institute for Health II - Task Force for Infectious Diseases Infectious Disease Epidemiology, Surveillance and Modelling Unit (GI-TFI2), Oberschleißheim, Germany

Electronic Supplementary Material

Table S1: Mutually adjusted odds ratios (OR) with 95% confidence intervals (CI) for death from COVID-19 compared with other non-external causes of death between March 2020 and July 2022 in Bavaria, stratified by sex and age, according to the official cause of death statistics.

|  | | **Men** | | | **Women** | | | **Under 65 years** | | | **65 to 74 years** | | | **75 to 84 years** | | | **85 years or older** | | |
| --- | --- | --- | --- | --- | --- | --- | --- | --- | --- | --- | --- | --- | --- | --- | --- | --- | --- | --- | --- |
| **ICD-10 code** | | **OR** | **95% CI** | | **OR** | **95% CI** | | **OR** | **95% CI** | | **OR** | **95% CI** | | **OR** | **95% CI** | | **OR** | **95% CI** | |
| **Sex (ref. women)** |  |  |  |  |  |  |  | 1.38 | 1.26 | 1.53 | 1.17 | 1.08 | 1.26 | 1.20 | 1.15 | 1.26 | 1.21 | 1.16 | 1.26 |
| **Age** |  |  |  |  |  |  |  |  |  |  |  |  |  |  |  |  |  |  |  |
| **Under 65 years (ref.)** |  |  |  |  |  |  |  |  |  |  |  |  |  |  |  |  |  |  |  |
| **65 to 74 years** |  | 1.16 | 1.08 | 1.24 | 1.35 | 1.22 | 1.49 |  |  |  |  |  |  |  |  |  |  |  |  |
| **75 to 84 years** |  | 1.39 | 1.30 | 1.48 | 1.59 | 1.46 | 1.74 |  |  |  |  |  |  |  |  |  |  |  |  |
| **85 years or older** |  | 1.37 | 1.29 | 1.46 | 1.58 | 1.45 | 1.72 |  |  |  |  |  |  |  |  |  |  |  |  |
| **Obesity** | **E66** | 1.85 | 1.62 | 2.10 | 2.44 | 2.16 | 2.76 | 3.04 | 2.59 | 3.57 | 2.05 | 1.73 | 2.43 | 1.67 | 1.40 | 1.99 | 1.28 | 0.98 | 1.67 |
| **Other degenerative diseases of nervous system (a.o. Alzheimer)** | **G30-G31** | 1.45 | 1.23 | 1.70 | 1.44 | 1.26 | 1.65 | 1.43 | 0.53 | 3.90 | 1.22 | 0.78 | 1.89 | 1.68 | 1.44 | 1.96 | 1.27 | 1.09 | 1.47 |
| **Dementia** | **F00-F03** | 1.66 | 1.57 | 1.76 | 1.74 | 1.65 | 1.82 | 1.92 | 1.19 | 3.07 | 2.04 | 1.73 | 2.41 | 1.78 | 1.67 | 1.90 | 1.63 | 1.56 | 1.71 |
| **Renal failure** | **N17-N19** | 1.56 | 1.49 | 1.64 | 1.55 | 1.47 | 1.63 | 2.46 | 2.16 | 2.80 | 2.09 | 1.90 | 2.30 | 1.58 | 1.49 | 1.68 | 1.34 | 1.27 | 1.41 |
| **Chronic lower respiratory diseases** | **J40-J47** | 1.26 | 1.18 | 1.35 | 1.43 | 1.32 | 1.54 | 1.43 | 1.21 | 1.70 | 1.29 | 1.15 | 1.45 | 1.37 | 1.26 | 1.49 | 1.28 | 1.17 | 1.39 |
| **Atrial fibrillation and flutter** | **I48** | 1.10 | 1.04 | 1.16 | 1.10 | 1.03 | 1.17 | 1.27 | 0.98 | 1.66 | 1.00 | 0.86 | 1.15 | 1.08 | 1.01 | 1.16 | 1.13 | 1.06 | 1.19 |
| **Ischaemic heart diseases** | **I20-I25** | 1.10 | 1.05 | 1.16 | 1.10 | 1.03 | 1.17 | 0.77 | 0.65 | 0.91 | 1.02 | 0.92 | 1.14 | 1.10 | 1.03 | 1.18 | 1.17 | 1.11 | 1.25 |
| **Diabetes mellitus** | **E10-E14** | 1.22 | 1.15 | 1.29 | 1.19 | 1.12 | 1.26 | 1.44 | 1.24 | 1.68 | 1.33 | 1.20 | 1.48 | 1.21 | 1.14 | 1.30 | 1.11 | 1.05 | 1.19 |
| **Hypertensive diseases** | **I10-I15** | 1.06 | 1.01 | 1.11 | 1.06 | 1.01 | 1.11 | 1.32 | 1.15 | 1.51 | 1.23 | 1.11 | 1.36 | 1.01 | 0.95 | 1.08 | 1.03 | 0.98 | 1.08 |
| **Malignant neoplasms*** | **C00-C97** | 1.17 | 1.10 | 1.24 | 1.20 | 1.11 | 1.29 | 1.97 | 1.68 | 2.31 | 1.59 | 1.42 | 1.78 | 1.19 | 1.11 | 1.28 | 0.94 | 0.86 | 1.01 |
| **Cerebrovascular diseases** | **I60-I69** | 1.08 | 1.01 | 1.15 | 0.97 | 0.90 | 1.05 | 1.15 | 0.93 | 1.43 | 1.24 | 1.08 | 1.42 | 1.07 | 0.99 | 1.16 | 0.92 | 0.86 | 1.00 |

* without secondary neoplasms

Source: own table

Table S2: Mutually adjusted odds ratios (ORs) with 95% confidence intervals (CI) for death from COVID-19 compared with other non-external causes of death between March 2020 and July 2022 in Bavaria according to the official cause of death statistics, stratified by pandemic wave in agreement with the retrospective phase classification of the Robert Koch Institute [2].

|  | | **All** | | | **Wave 1 02/03/2020 - 17/05/2020** | | | **Wave 2 28/09/2020 - 28/02/2021** | | | **Wave 3 01/03/2021 - 13/06/2021** | | | **Wave 4 02/08/2021 - 26/12/2021** | | | **Wave 5 27/12/2021 - 29/05/2022** | | |
| --- | --- | --- | --- | --- | --- | --- | --- | --- | --- | --- | --- | --- | --- | --- | --- | --- | --- | --- | --- |
| **ICD-10 code** | | **OR** | **95% CI** | | **OR** | **95% CI** | | **OR** | **95% CI** | | **OR** | **95% CI** | | **OR** | **95% CI** | | **OR** | **95% KI** | |
| **Sex (ref. women)** |  | 1.22 | 1.18 | 1.25 | 1.33 | 1.22 | 1.46 | 1.12 | 1.07 | 1.18 | 1.29 | 1.19 | 1.41 | 1.31 | 1.23 | 1.39 | 1.20 | 1.12 | 1.28 |
| **Age** |  |  |  |  |  |  |  |  |  |  |  |  |  |  |  |  |  |  |  |
| **Under 65 years (ref.)** |  |  |  |  |  |  |  |  |  |  |  |  |  |  |  |  |  |  |  |
| **65 to 74 years** |  | 1.22 | 1.15 | 1.29 | 1.35 | 1.11 | 1.64 | 1.62 | 1.44 | 1.81 | 1.24 | 1.08 | 1.43 | 0.95 | 0.85 | 1.07 | 1.11 | 0.97 | 1.28 |
| **75 to 84 years** |  | 1.45 | 1.38 | 1.53 | 1.75 | 1.47 | 2.08 | 2.32 | 2.10 | 2.57 | 0.98 | 0.86 | 1.12 | 1.05 | 0.95 | 1.16 | 1.33 | 1.18 | 1.50 |
| **85 years or older** |  | 1.44 | 1.37 | 1.52 | 1.55 | 1.30 | 1.84 | 2.49 | 2.25 | 2.75 | 0.65 | 0.57 | 0.75 | 0.92 | 0.83 | 1.01 | 1.48 | 1.31 | 1.67 |
| **Obesity** | **E66** | 2.12 | 1.94 | 2.32 | 1.74 | 1.28 | 2.37 | 1.60 | 1.33 | 1.91 | 2.64 | 2.13 | 3.26 | 3.02 | 2.54 | 3.59 | 1.75 | 1.38 | 2.22 |
| **Other degenerative diseases of nervous system (a.o. Alzheimer)** | **G30-G31** | 1.44 | 1.30 | 1.60 | 1.42 | 1.03 | 1.97 | 1.53 | 1.30 | 1.80 | 1.18 | 0.73 | 1.91 | 1.32 | 1.00 | 1.75 | 1.58 | 1.25 | 1.98 |
| **Dementia** | **F00-F03** | 1.71 | 1.64 | 1.77 | 1.65 | 1.46 | 1.86 | 2.03 | 1.91 | 2.15 | 0.89 | 0.75 | 1.04 | 1.25 | 1.14 | 1.38 | 1.94 | 1.78 | 2.11 |
| **Renal failure** | **N17-N19** | 1.56 | 1.50 | 1.61 | 1.62 | 1.45 | 1.82 | 1.61 | 1.51 | 1.70 | 1.75 | 1.57 | 1.95 | 1.62 | 1.49 | 1.75 | 1.38 | 1.26 | 1.50 |
| **Chronic lower respiratory diseases** | **J40-J47** | 1.33 | 1.26 | 1.40 | 1.40 | 1.20 | 1.65 | 1.41 | 1.30 | 1.54 | 1.24 | 1.06 | 1.45 | 1.24 | 1.10 | 1.40 | 1.39 | 1.23 | 1.57 |
| **Atrial fibrillation and flutter** | **I48** | 1.10 | 1.05 | 1.14 | 1.15 | 1.01 | 1.31 | 1.06 | 0.99 | 1.14 | 1.17 | 1.02 | 1.34 | 1.11 | 1.00 | 1.22 | 1.12 | 1.02 | 1.24 |
| **Ischaemic heart diseases** | **I20-I25** | 1.10 | 1.06 | 1.14 | 1.24 | 1.10 | 1.40 | 1.13 | 1.05 | 1.21 | 1.08 | 0.95 | 1.22 | 1.10 | 1.00 | 1.20 | 0.93 | 0.84 | 1.03 |
| **Diabetes mellitus** | **E10-E14** | 1.21 | 1.16 | 1.26 | 1.20 | 1.05 | 1.36 | 1.21 | 1.14 | 1.30 | 1.18 | 1.04 | 1.35 | 1.23 | 1.12 | 1.35 | 1.13 | 1.03 | 1.25 |
| **Hypertensive diseases** | **I10-I15** | 1.06 | 1.03 | 1.10 | 1.30 | 1.17 | 1.45 | 1.08 | 1.01 | 1.14 | 1.19 | 1.06 | 1.33 | 0.99 | 0.91 | 1.08 | 0.92 | 0.84 | 1.01 |
| **Malignant neoplasms*** | **C00-C97** | 1.18 | 1.12 | 1.24 | 1.17 | 1.01 | 1.37 | 1.07 | 0.99 | 1.16 | 1.23 | 1.06 | 1.42 | 1.02 | 0.91 | 1.15 | 1.47 | 1.33 | 1.63 |
| **Cerebrovascular diseases** | **I60-I69** | 1.03 | 0.98 | 1.08 | 1.05 | 0.90 | 1.23 | 1.08 | 0.99 | 1.17 | 0.90 | 0.76 | 1.07 | 0.90 | 0.80 | 1.02 | 1.10 | 0.98 | 1.23 |

* without secondary neoplasms

Source: own table

**Table S3: Most frequent comorbidities and previous diseases of persons ≥ 65 years with COVID-19 as underlying cause of death during the pandemic waves 1 to 5 in agreement with the retrospective phase classification of the Robert Koch Institute [2] in Bavaria (in percent)** **according to the official cause of death statistics.**

|  | **Wave 1 02/03/2020 - 17/05/2020** | **Wave 2 28/09/2020 - 28/02/2021** | **Wave 3 01/03/2021 - 13/06/2021** | **Wave 4 02/08/2021 - 26/12/2021** | **Wave 5 27/12/2021 - 29/05/2022** |
| --- | --- | --- | --- | --- | --- |
|  |  |  |  |  |  |
| **Dementia (F00 – F03)** | 18.9 | 23.0 | 9.1 | 14.0 | 21.8 |
| **Renal failure (N17 – N19)** | 19.9 | 19.4 | 20.6 | 18.8 | 17.3 |
| **Hypertensive diseases (I10 – I15)** | 22.9 | 19.8 | 19.3 | 17.5 | 16.8 |
| **Ischaemic heart diseases**  **(I20 – I25)** | 16.2 | 13.6 | 15.0 | 13.5 | 11.6 |
| **Diabetes mellitus (E10 – E14)** | 15.2 | 13.8 | 14.2 | 13.1 | 12.4 |
| **Atrial fibrillation and flutter (I48)** | 13.5 | 12.1 | 12.5 | 11.8 | 12.7 |
| **Malignant neoplasms***  **(C00 – C97)** | 8.8 | 7.7 | 9.3 | 7.8 | 10.5 |
| **Cerebrovascular diseases**  **(I60-I69)** | 8.4 | 8.6 | 6.5 | 6.7 | 8.3 |
| **Chronic lower respiratory**  **diseases (J40-J47)** | 7.9 | 7.1 | 8.0 | 7.5 | 7.3 |

* without secondary neoplasms

Source: own table

**Table S4: Most frequent comorbidities and previous diseases of persons ≥ 65 years with COVID-19 as underlying cause of death during the pandemic waves 1 to 5 (alternative phase classification) in Bavaria (in percent)** **according to the official cause of death statistics.**

|  | **Wave 1 02/03/2020 - 31/05/2020** | **Wave 2 02/11/2020 - 28/02/2021** | **Wave 3 01/03/2021 - 30/05/2021** | **Wave 4 04/10/2021 - 26/12/2021** | **Wave 5 27/12/2021 - 29/05/2022** |
| --- | --- | --- | --- | --- | --- |
|  |  |  |  |  |  |
| **Dementia (F00 – F03)** | 19.1 | 23.0 | 8.9 | 14.0 | 22.1 |
| **Renal failure (N17 – N19)** | 20.1 | 19.4 | 20.5 | 18.8 | 17.3 |
| **Hypertensive diseases (I10 – I15)** | 22.7 | 19.9 | 18.9 | 17.5 | 16.6 |
| **Ischaemic heart diseases**  **(I20 – I25)** | 16.2 | 13.6 | 15.2 | 13.6 | 11.6 |
| **Diabetes mellitus (E10 – E14)** | 15.1 | 13.8 | 14.1 | 12.9 | 12.5 |
| **Atrial fibrillation and flutter (I48)** | 13.6 | 12.1 | 12.6 | 12.0 | 12.7 |
| **Malignant neoplasms***  **(C00 – C97)** | 8.7 | 7.7 | 9.4 | 7.9 | 10.5 |
| **Cerebrovascular diseases**  **(I60-I69)** | 8.4 | 8.6 | 6.7 | 6.7 | 8.4 |
| **Chronic lower respiratory**  **diseases (J40-J47)** | 7.8 | 7.0 | 8.0 | 7.5 | 7.4 |

* without secondary neoplasms

Source: own table

**Table S5: Most frequent comorbidities and previous diseases of persons ≥ 65 years with non-external underlying causes of death other than COVID-19 during the pandemic waves 1 to 5 in agreement with the retrospective phase classification of the Robert Koch Institute [2] in Bavaria (in percent)** **according to the official cause of death statistics.**

|  | **Wave 1 02/03/2020 - 17/05/2020** | **Wave 2 28/09/2020 - 28/02/2021** | **Wave 3 01/03/2021 - 13/06/2021** | **Wave 4 02/08/2021 - 26/12/2021** | **Wave 5 27/12/2021 - 29/05/2022** |
| --- | --- | --- | --- | --- | --- |
|  |  |  |  |  |  |
| **Dementia (F00 – F03)** | 12.5 | 12.2 | 11.6 | 11.8 | 12.1 |
| **Renal failure (N17 – N19)** | 12.4 | 12.8 | 13.2 | 12.4 | 12.9 |
| **Hypertensive diseases (I10 – I15)** | 17.3 | 16.8 | 16.5 | 16.5 | 16.7 |
| **Ischaemic heart diseases**  **(I20 – I25)** | 11.5 | 11.6 | 11.6 | 10.9 | 11.2 |
| **Diabetes mellitus (E10 – E14)** | 11.1 | 10.5 | 10.7 | 10.1 | 10.4 |
| **Atrial fibrillation and flutter (I48)** | 10.3 | 10.1 | 10.5 | 10.0 | 10.6 |
| **Malignant neoplasms***  **(C00 – C97)** | 7.6 | 7.6 | 7.6 | 7.5 | 7.8 |
| **Cerebrovascular diseases**  **(I60-I69)** | 7.2 | 7.5 | 7.4 | 7.3 | 7.3 |
| **Chronic lower respiratory**  **diseases (J40-J47)** | 5.6 | 5.3 | 5.7 | 5.4 | 5.3 |

* without secondary neoplasms

Source: own table

**Table S6: Most frequent comorbidities and previous diseases of persons ≥ 65 years with non-external underlying causes of death other than COVID-19 during the pandemic waves 1 to 5 (alternative phase classification) in Bavaria (in percent)** **according to the official cause of death statistics.**

|  | **Wave 1 02/03/2020 - 31/05/2020** | **Wave 2 02/11/2020 - 28/02/2021** | **Wave 3 01/03/2021 - 30/05/2021** | **Wave 4 04/10/2021 - 26/12/2021** | **Wave 5 27/12/2021 - 29/05/2022** |
| --- | --- | --- | --- | --- | --- |
|  |  |  |  |  |  |
| **Dementia (F00 – F03)** | 12.3 | 12.2 | 11.6 | 11.7 | 12.2 |
| **Renal failure (N17 – N19)** | 12.6 | 12.8 | 13.2 | 12.3 | 12.9 |
| **Hypertensive diseases (I10 – I15)** | 17.1 | 16.8 | 16.6 | 16.6 | 16.8 |
| **Ischaemic heart diseases**  **(I20 – I25)** | 11.5 | 11.6 | 11.7 | 10.8 | 11.2 |
| **Diabetes mellitus (E10 – E14)** | 10.9 | 10.6 | 10.8 | 10.2 | 10.5 |
| **Atrial fibrillation and flutter (I48)** | 10.3 | 10.1 | 10.5 | 10.1 | 10.6 |
| **Malignant neoplasms***  **(C00 – C97)** | 7.7 | 7.5 | 7.6 | 7.4 | 7.8 |
| **Cerebrovascular diseases**  **(I60-I69)** | 7.1 | 7.4 | 7.5 | 7.2 | 7.2 |
| **Chronic lower respiratory**  **diseases (J40-J47)** | 5.7 | 5.3 | 5.7 | 5.4 | 5.3 |

* without secondary neoplasms

Source: own table

Table S7: Average marginal effects (AME, in percent) and confidence intervalls (CI) for death from COVID-19 compared with other non-external causes of death between March 2020 and July 2022 in Bavaria, stratified by sex and age, according to the official cause of death statistics.

|  |  | **Men** | | **Women** | | **Under 65 years** | | **65 to 74 years** | | **75 to 84 years** | | **85 years or older** | |
| --- | --- | --- | --- | --- | --- | --- | --- | --- | --- | --- | --- | --- | --- |
|  | **ICD-10 Code** | **AME** | **95% CI**  **-/+** | **AME** | **95% CI**  **-/+** | **AME** | **95% CI**  **-/+** | **AME** | **95% CI**  **-/+** | **AME** | **95% CI**  **-/+** | **AME** | **95% CI**  **-/+** |
| **Sex (ref. women)** |  |  |  |  |  | 1.53405 | 0.00762 | 0.90033 | 0.00310 | 1.31716 | 0.00233 | 1.35899 | 0.00169 |
| **Age** |  |  |  |  |  |  |  |  |  |  |  |  |  |
| **Under 65 years (ref.)** |  |  |  |  |  |  |  |  |  |  |  |  |  |
| **65 to 74 years** |  | 1.01970 | 0.00141 | 1.89480 | 0.00297 |  |  |  |  |  |  |  |  |
| **75 to 84 years** |  | 2.31818 | 0.00321 | 2.93828 | 0.00461 |  |  |  |  |  |  |  |  |
| **85 years or older** |  | 2.25660 | 0.00312 | 2.87555 | 0.00451 |  |  |  |  |  |  |  |  |
|  |  |  |  |  |  |  |  |  |  |  |  |  |  |
| **Obesity** | **E66** | 4.33759 | 0.00600 | 5.58969 | 0.00877 | 5.23076 | 0.02600 | 4.24857 | 0.01464 | 3.66149 | 0.00645 | 1.75707 | 0.00219 |
| **Other degenerative diseases of nervous system (a.o. Alzheimer)** | **G30-G31** | 2.60503 | 0.00360 | 2.28367 | 0.00358 | 1.68758 | 0.00839 | 1.17728 | 0.00406 | 3.70572 | 0.00653 | 1.67924 | 0.00209 |
| **Dementia** | **F00-F03** | 3.61462 | 0.00500 | 3.45938 | 0.00543 | 3.06716 | 0.01525 | 4.22368 | 0.01455 | 4.15594 | 0.00732 | 3.49030 | 0.00435 |
| **Renal failure** | **N17-N19** | 3.16311 | 0.00438 | 2.74223 | 0.00430 | 4.23110 | 0.02103 | 4.37708 | 0.01508 | 3.30276 | 0.00582 | 2.08304 | 0.00259 |
| **Chronic lower respiratory diseases** | **J40-J47** | 1.64582 | 0.00228 | 2.23592 | 0.00351 | 1.68848 | 0.00839 | 1.52596 | 0.00525 | 2.26079 | 0.00399 | 1.74596 | 0.00217 |
| **Atrial fibrillation and flutter** | **I48** | 0.65592 | 0.00091 | 0.57360 | 0.00090 | 1.11214 | 0.00553 | -0.02736 | 0.00009 | 0.56621 | 0.00100 | 0.85288 | 0.00106 |
| **Ischaemic heart diseases** | **I20-I25** | 0.68159 | 0.00095 | 0.57559 | 0.00090 | -1.23839 | 0.00616 | 0.13284 | 0.00045 | 0.70897 | 0.00125 | 1.14487 | 0.00142 |
| **Diabetes mellitus** | **E10-E14** | 1.41038 | 0.00195 | 1.06985 | 0.00168 | 1.72795 | 0.00859 | 1.68041 | 0.00579 | 1.39443 | 0.00246 | 0.76775 | 0.00096 |
| **Hypertensive diseases** | **I10-I15** | 0.40794 | 0.00056 | 0.37886 | 0.00060 | 1.29213 | 0.00642 | 1.22641 | 0.00423 | 0.09029 | 0.00016 | 0.21374 | 0.00027 |
| **Malignant neoplasms*** | **C00-C97** | 1.08774 | 0.00151 | 1.12992 | 0.00177 | 3.18926 | 0.01586 | 2.74676 | 0.00946 | 1.25665 | 0.00222 | -0.47090 | 0.00059 |
| **Cerebrovascular diseases** | **I60-I69** | 0.52924 | 0.00073 | -0.19156 | 0.00030 | 0.65991 | 0.00328 | 1.25509 | 0.00432 | 0.46954 | 0.00082 | -0.58079 | 0.00072 |

* without secondary neoplasms

Source: own table

**Table S8: Average marginal effects (AME, in percent) and confidence intervalls (CI) for death from COVID-19 compared with other non-external causes of death between March 2020 and July 2022 in Bavaria according to the official cause of death statistics, stratified by pandemic wave in agreement with the retrospective phase classification of the Robert Koch Institute [2].**

|  | | **All** | | **Wave 1 02/03/2020 - 17/05/2020** | | **Wave 2 28/09/2020 - 28/02/2021** | | **Wave 3 01/03/2021 - 13/06/2021** | | **Wave 4 02/08/2021 - 26/12/2021** | | **Wave 5 27/12/2021 - 29/05/2022** | |
| --- | --- | --- | --- | --- | --- | --- | --- | --- | --- | --- | --- | --- | --- |
|  | **ICD-10 code** | **AME** | **95% CI**  **-/+** | **AME** | **95% CI**  **-/+** | **AME** | **95% CI**  **-/+** | **AME** | **95% CI**  **-/+** | **AME** | **95% CI**  **-/+** | **AME** | **95% CI**  **-/+** |
| **Sex (ref. women)** |  | 1.29747 | 0.00137 | 1.99749 | 0.00825 | 1.40932 | 0.00348 | 1.51562 | 0.00612 | 1.95751 | 0.00442 | 1.15637 | 0.00306 |
| **Age** |  |  |  |  |  |  |  |  |  |  |  |  |  |
| **Under 65 years (ref.)** |  |  |  |  |  |  |  |  |  |  |  |  |  |
| **65 to 74 years** |  | 1.31043 | 0.00138 | 2.07914 | 0.00859 | 5.80929 | 0.01436 | 1.27152 | 0.00514 | -0.37330 | 0.00084 | 0.69886 | 0.00185 |
| **75 to 84 years** |  | 2.49848 | 0.00264 | 3.89558 | 0.01609 | 10.18888 | 0.02519 | -0.10755 | 0.00044 | 0.33262 | 0.00076 | 1.85349 | 0.00490 |
| **85 years or older** |  | 2.43601 | 0.00257 | 3.03654 | 0.01254 | 11.03515 | 0.02728 | -2.51236 | 0.01015 | -0.64686 | 0.00146 | 2.53814 | 0.00671 |
|  |  |  |  |  |  |  |  |  |  |  |  |  |  |
| **Obesity** | **E66** | 5.02381 | 0.00531 | 3.85033 | 0.01590 | 5.65218 | 0.01397 | 5.73122 | 0.02313 | 8.07940 | 0.01824 | 3.58725 | 0.00948 |
| **Other degenerative diseases of nervous system (a.o. Alzheimer)** | **G30-G31** | 2.44179 | 0.00258 | 2.45656 | 0.01015 | 5.13999 | 0.01270 | 0.95836 | 0.00387 | 2.02366 | 0.00457 | 2.91029 | 0.00770 |
| **Dementia** | **F00-F03** | 3.56266 | 0.00377 | 3.49075 | 0.01442 | 8.56838 | 0.02118 | -0.71393 | 0.00288 | 1.63622 | 0.00369 | 4.24127 | 0.01121 |
| **Renal failure** | **N17-N19** | 2.95330 | 0.00312 | 3.36290 | 0.01390 | 5.72940 | 0.01416 | 3.31651 | 0.01339 | 3.51346 | 0.00793 | 2.07234 | 0.00548 |
| **Chronic lower respiratory diseases** | **J40-J47** | 1.90751 | 0.00202 | 2.35172 | 0.00971 | 4.19542 | 0.01037 | 1.26268 | 0.00509 | 1.57550 | 0.00355 | 2.13278 | 0.00564 |
| **Atrial fibrillation and flutter** | **I48** | 0.61187 | 0.00064 | 0.96327 | 0.00398 | 0.74746 | 0.00185 | 0.93060 | 0.00376 | 0.72695 | 0.00164 | 0.71936 | 0.00190 |
| **Ischaemic heart diseases** | **I20-I25** | 0.62684 | 0.00066 | 1.50232 | 0.00621 | 1.44958 | 0.00359 | 0.45012 | 0.00182 | 0.67601 | 0.00153 | -0.42907 | 0.00114 |
| **Diabetes mellitus** | **E10-E14** | 1.24746 | 0.00132 | 1.23678 | 0.00511 | 2.35145 | 0.00581 | 0.99750 | 0.00403 | 1.52064 | 0.00344 | 0.82983 | 0.00219 |
| **Hypertensive diseases** | **I10-I15** | 0.39708 | 0.00042 | 1.82231 | 0.00753 | 0.87677 | 0.00217 | 1.02869 | 0.00416 | -0.05983 | 0.00014 | -0.50910 | 0.00134 |
| **Malignant neoplasms*** | **C00-C97** | 1.09495 | 0.00115 | 1.10612 | 0.00457 | 0.82746 | 0.00205 | 1.21816 | 0.00492 | 0.17600 | 0.00040 | 2.46288 | 0.00651 |
| **Cerebrovascular diseases** | **I60-I69** | 0.17583 | 0.00019 | 0.34630 | 0.00143 | 0.90846 | 0.00225 | -0.63200 | 0.00255 | -0.74964 | 0.00169 | 0.57467 | 0.00152 |

* without secondary neoplasms

Source: own table
